# A Comparative Bioinformatics Analysis of the Transcriptomic Profiles of Peri-Implantitis and Periodontitis and Their Common Signaling Pathways with Atherosclerosis

**DOI:** 10.3390/cimb48040401

**Published:** 2026-04-14

**Authors:** Aleksandr V. Guskov, Anatoliy S. Utyuzh, Aleksandr A. Oleynikov, Aleksandr A. Nikiforov, Stanislav N. Kotlyarov

**Affiliations:** 1 Department of Orthopedic Dentistry and Orthodontics, Ryazan State Medical University, 390026 Ryazan, Russia; guskov74@gmail.com (A.V.G.);; 2 Department of Orthopedic Dentistry, N.A. Semashko National Research Institute of Public Health, 105064 Moscow, Russia; 3 Department of Pharmacology, Ryazan State Medical University, 390026 Ryazan, Russia; 4 Department of Nursing, Ryazan State Medical University, 390026 Ryazan, Russia

**Keywords:** peri-implantitis, periodontitis, atherosclerosis, transcriptomic analysis, bioinformatics, differentially expressed genes, immune inflammation, immune deconvolution

## Abstract

(1) Objective. To conduct a comparative bioinformatics analysis of the transcriptomic profiles of peri-implantitis and periodontitis to identify common and specific molecular signatures underlying their pathogenesis, as well as molecular parallels with atherosclerosis. (2) Methods: We used datasets from the Gene Expression Omnibus (GEO) database: dataset GSE223924 (30 gingival tissue samples from patients with peri-implantitis, periodontitis, and healthy subjects) and GSE100927 (atherosclerotic and control tissue; n = 104). Differentially expressed genes (DEGs) were identified based on the criteria: |logFC| > 1 and FDR < 0.05. To quantitatively assess the relative abundance of immune cells, we used the xCell deconvolution algorithm. (3) Results: In the peri-implantitis group, 3669 DEGs with upregulated expression and 3106 with downregulated expression were identified; in the periodontitis group, 1968 and 1250 DEGs, respectively. Functional analysis of the upregulated DEGs revealed activation of inflammatory processes, cell adhesion, and angiogenesis in both diseases. Key differences lay in the activation of adaptive immune mechanisms in peri-implantitis (enrichment of the “graft rejection” and “T-cell receptor signaling”) and innate immunity in periodontitis (enrichment of the “lipopolysaccharide response” and “Toll-like receptors (TLR) signaling” pathways). Analysis of downregulated DEGs revealed more profound disruptions in cytoskeletal organization and epithelial differentiation in periodontitis, as well as suppression of xenobiotic and lipid metabolism in both diseases. xCell deconvolution confirmed a significant increase in B cells, neutrophils, monocytes, M1 macrophages, and dendritic cells in peri-implantitis, and also revealed a trend toward an increase in these cells in periodontitis (*p* > 0.05), which is consistent with the activation of TLR signaling. In periodontitis, a significant increase in M2 macrophages and a decrease in Th1 cells were observed. Comparison with atherosclerosis revealed 272 common DEGs with peri-implantitis and 173 common DEGs with periodontitis. Functional analysis of the common genes confirmed their role in leukocyte transendothelial migration, cytokine production, and the “Lipids and Atherosclerosis” pathway. (4) Conclusions: Functional analysis and immune deconvolution consistently demonstrate that peri-implantitis is characterized by statistically significant activation of both adaptive and innate immunity, whereas in periodontitis, the activation of innate immunity manifests primarily at the level of signaling pathways. The significant overlap found between the transcriptional profiles of both diseases and atherosclerosis may indicate the presence of common pathogenetic links.

## 1. Introduction

Inflammatory periodontal diseases, such as periodontitis and peri-implantitis, represent one of the most pressing challenges in modern dentistry. Periodontitis, an infectious inflammatory disease of the periodontal tissues, is one of the leading causes of tooth loss in the adult population. According to global epidemiological studies, severe forms of periodontitis affect up to 10–15% of the adult population [1,2,3]. Peri-implantitis, an inflammatory disease of the tissues surrounding dental implants, represents an equally important clinical problem. It complicates the management of these patients and may affect treatment outcomes and quality of life [4,5,6,7].

Despite a similar clinical presentation, which includes soft tissue inflammation, bleeding, and progressive bone resorption, periodontitis and peri-implantitis exhibit certain differences. Peri-implantitis is characterized by faster progression, more severe tissue destruction, and a poorer response to standard periodontal treatment compared to periodontitis [8,9,10]. The reasons for these differences have not yet been fully elucidated, but it is believed that they may be related to the characteristics of osteoimmune regulation [11,12,13,14].

The pathogenesis of periodontitis has been studied in considerable detail. The disease develops as a result of complex interactions between microorganisms in the oral cavity and the host’s immune system. Periodontopathogenic bacteria (*Porphyromonas gingivalis*, *Tannerella forsythia*, *Treponema denticola*, and others) activate innate immune receptors (Toll-like receptors (TLRs)), which triggers a cascade of pro-inflammatory cytokines (interleukin (IL)-1β, tumor necrosis factor alpha (TNF-α), IL-6) and chemokines (IL-8) [15,16,17]. This leads to the recruitment of neutrophils, macrophages, and lymphocytes to the site of inflammation. Disease progression is associated with an imbalance between pro- and anti-inflammatory mechanisms, a predominance of T-helper responses (Th1, Th17), and the activation of osteoclastogenesis *via* receptor activator of nuclear factor-kappa B ligand (RANKL)-dependent pathways [18,19,20].

The pathogenesis of peri-implantitis is also an area of intensive research. Histological data indicate differences in the cellular composition of the inflammatory infiltrate in peri-implantitis compared to periodontitis [10,21,22]. These and other observations suggest that the immune mechanisms of the two diseases may differ, which is a relevant and promising area of research.

The issue of inflammatory periodontal diseases takes on particular significance in light of their proven association with systemic diseases, particularly cardiovascular disease [7,23,24]. Numerous epidemiological studies have demonstrated that in patients with periodontitis, the risk of developing atherosclerosis and related cardiovascular events (myocardial infarction, stroke) is increased by 20–40% [25,26,27]. Meta-analyses confirm that periodontitis is an independent risk factor for cardiovascular disease [28,29,30]. Evidence is also beginning to accumulate on the links between peri-implantitis and cardiovascular diseases [31,32,33].

Several hypotheses have been proposed to explain the link between inflammation in periodontal tissues and atherosclerosis. Systemic inflammation is considered a leading mechanism. Pro-inflammatory cytokines (IL-1β, TNF-α, IL-6) produced at sites of periodontal inflammation enter the systemic circulation and sustain chronic low-grade inflammation in the vascular wall. The role of direct bacterial invasion is also being studied, whereby periodontal pathogens and their components (lipopolysaccharide (LPS), DNA) can enter the bloodstream, reach atherosclerotic plaques, and directly contribute to their progression. However, the molecular basis of this association remains poorly understood. In particular, it is unclear exactly which genes and signaling pathways are common to inflammatory periodontal diseases and atherosclerosis. These data are of clinical interest, as they could potentially be used as biomarkers of disease progression or in assessing treatment efficacy, as well as, in the future, as therapeutic targets.

The aim of this study is to conduct a comprehensive comparative bioinformatics analysis of the transcriptomic profiles of peri-implantitis and periodontitis to identify common and specific molecular signatures of their pathogenesis, as well as molecular parallels with atherosclerosis.

## 2. Materials and Methods

### 2.1. Data Sources and Preprocessing

To identify common molecular signatures between atherosclerosis and inflammatory periodontal diseases (periodontitis and peri-implantitis), a comparative transcriptomic analysis was performed using data from the Gene Expression Omnibus (NCBI) database. Datasets were selected using the keywords “Periodontitis,” “Peri-implantitis,” and “Atherosclerosis.”

Periodontal disease dataset:

The GSE223924 dataset was used, containing gene expression profiles in gingival tissue from 20 patients: 10 healthy subjects and 10 patients with periodontitis and peri-implantitis (10 healthy, 10 periodontitis, and 10 peri-implantitis tissues). Data were obtained on the Illumina NovaSeq 6000 platform (Homo sapiens) (GPL24676). Normalization was performed using the quartile normalization method followed by log2 transformation using the Phantasus platform (v.1.31.1) [34].

Atherosclerosis dataset:

The GSE100927 dataset was included for analysis, comprising transcriptomic profiles of 104 samples: 69 atherosclerotic lesions (carotid, femoral, and popliteal arteries) and 35 control samples of intact arteries. The data were acquired on the Agilent-039494 SurePrint G3 platform (GPL17077) and normalized using the LOWESS method [35]. Normalization was performed by the dataset authors using the LOWESS method; no additional preprocessing was conducted.

### 2.2. Identification of Differentially Expressed Genes (DEGs)

Differential expression analysis was performed using the built-in tools of the Phantasus platform (v.1.31.1), which implements the algorithms of the limma package in the R environment. For all pairwise comparisons (periodontitis vs. control, peri-implantitis vs. control, atherosclerosis vs. control), identical cutoff criteria were applied: absolute log_2_ fold change (|logFC|) > 1 and adjusted *p*-value (false discovery rate, FDR) < 0.05. Multiple comparisons were corrected using the Benjamini & Hochberg method.

For each dataset, comparison groups were formed: peri-implantitis vs. control, periodontitis vs. control, and atherosclerosis vs. control. Then, using the identified DEGs, common differentially expressed genes were identified in the peri-implantitis–atherosclerosis and periodontitis–atherosclerosis groups. The intersecting sets of DEGs were visualized using Venn diagrams generated in Funrich (v 3.1.3).

### 2.3. Functional and Network Analysis

Functional annotation of DEG lists (separately for upregulated and downregulated DEGs) using Gene Ontology (Biological Process, BP) terms and Kyoto Encyclopedia of Genes and Genomes (KEGG) pathways was performed using ShinyGO 0.85.1 [36]. The significance level was set at *p* < 0.05. PPI networks were constructed for all DEGs (logFC > 1, FDR < 0.05) for each disease using the STRING database (v. 12.0) (minimum interaction confidence threshold ≥ 0.4). Network analysis and visualization were performed in Cytoscape (v.3.9.1). Key genes (hubs) in each network were identified using the cytoHubba plugin with the Maximal Clique Centrality (MCC) algorithm. The top 10 genes were selected for further analysis.

### 2.4. Cell Type Enrichment Analysis

The xCell method was used to quantify the relative abundance of immune cells. Deconvolution of the cellular composition was performed using the xCell v.1.1.0 package in R (v.4.2.1) [37]. Estimates of the relative abundance of 64 cell types, including T-cell subpopulations (CD4^+^ naïve, CD8^+^ Tcm, Treg, Th1, Th2), were obtained for each sample. Statistical significance of differences between groups was assessed using a two-sample *t*-test. Differences were considered statistically significant at *p* < 0.05.

### 2.5. Validation of the Diagnostic Significance of Genes

To validate the identified key hub genes, independent datasets that were not used at the DEG identification stage were employed. This approach allowed objective assessment of the diagnostic value of the genes without the risk of overfitting. For peri-implantitis, the GSE33774 dataset was used, comprising transcriptomic profiles of gingival tissues obtained on a microchip platform: healthy control, 8 samples; peri-implantitis, 7 samples [38]. For periodontitis, a large independent dataset with high statistical power, GSE10334 (183 periodontitis samples, 64 controls), was used [39].

ROC (Receiver Operating Characteristic) analysis was performed using R version 4.2.2 and the pROC package (version 1.18.0). The Mann–Whitney U test (for GSE33774) and the DeLong method (for GSE10334) were used to assess the statistical significance of differences in gene expression between groups (peri-implantitis vs. control; periodontitis vs. control). Differences were considered statistically significant at *p* < 0.05.

## 3. Results

### 3.1. Identification of DEGs in Peri-Implantitis and Periodontitis

A comparative analysis of the GSE223924 dataset revealed significant changes in gene expression profiles in peri-implantitis and periodontitis compared to the control group. In the peri-implantitis vs. control group, 3669 differentially expressed genes (DEGs) with upregulated expression and 3106 DEGs with downregulated expression were identified, while in the periodontitis vs. control group, 1968 DEGs with upregulated expression and 1250 DEGs with downregulated expression were identified.

### 3.2. Functional Analysis of DEGs in Peri-Implantitis and Periodontitis

#### 3.2.1. Upregulated Genes

**Figure 1:**
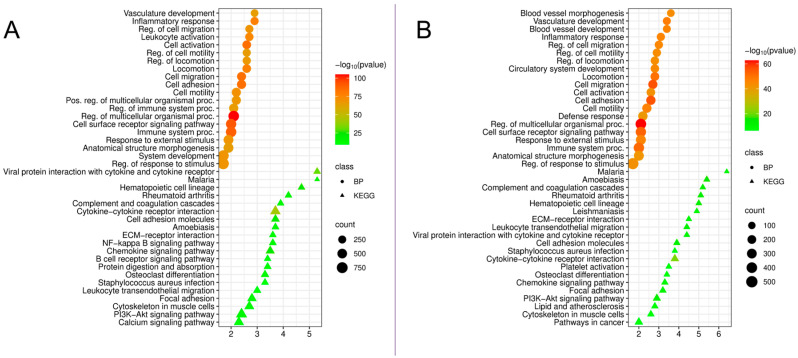
(**A**). Functional enrichment analysis by biological processes (BPs) and KEGG pathways of upregulated DEGs in peri-implantitis compared to the control; (**B**). Functional enrichment analysis based on biological processes and KEGG pathways of upregulated DEGs in periodontitis compared to controls.

Analysis of upregulated DEGs revealed marked activation of inflammatory and immune mechanisms in gingival tissues in both diseases, but with significant differences (Figure 1).

The most striking difference in periodontitis was the exceptionally high enrichment of processes related to the vascular system: “blood vessel morphogenesis” (enrichment = 3.6, *p* = 1.8 × 10^−44^), “vasculature development” (enrichment = 3.4, *p* = 2.6 × 10^−48^), and “blood vessel development” (enrichment = 3.4, *p* = 1.8 × 10^−46^). These processes are also present in peri-implantitis (“vasculature development,” enrichment = 2.9, *p* = 2.0 × 10^−65^), but are significantly lower in terms of enrichment. This may indicate more active angiogenesis and vascular remodeling in periodontitis, possibly associated with a larger area of involvement and the chronicity of the inflammatory process.

In both diseases, the processes “inflammatory response” (peri-implantitis—2.9, *p* = 1.8 × 10^−78^; periodontitis—3.1, *p* = 1.1 × 10^−46^), “regulation of cell migration” (2.7 and 3.0, respectively), and “cell adhesion” (2.4 and 2.6) show high enrichment. These data may reflect the recruitment of immune cells and their interaction with the endothelium and extracellular matrix.

The most significant difference between the two diseases was identified in the activation of adaptive humoral immunity. The B cell receptor signaling pathway showed significant enrichment only in peri-implantitis (3.4, *p* = 4.3 × 10^−10^) and was absent from the list of enriched pathways in periodontitis.

Analysis of KEGG pathways revealed both common and disease-specific mechanisms. Common pathways for both diseases include cytokine-cytokine receptor interaction—a key pathway of intercellular communication during inflammation (peri-implantitis: 3.7, *p* = 4.3 × 10^−43^; periodontitis: 3.8, *p* = 1.4 × 10^−21^); the chemokine signaling pathway, which reflects leukocyte recruitment (3.5 and 3.3, respectively); cell adhesion molecules–cell–microenvironment interactions (3.7 and 3.9); osteoclast differentiation indicates the activation of bone resorption (3.3 and 3.4); extracellular matrix (ECM)-receptor interaction and focal adhesion.

In peri-implantitis, several highly enriched pathways were observed that were either absent or less pronounced in periodontitis: the nuclear factor kappa‑B (NF‑κB) signaling pathway (3.6, *p* = 2.1 × 10^−14^), a central regulator of inflammation; the B cell receptor signaling pathway (3.4, *p* = 4.3 × 10^−10^), indicating the role of humoral immunity; phagosome pathway; and the phosphatidylinositol 3-kinase (PI3K)-Akt signaling pathway (2.4, *p* = 8.4 × 10^−16^), which regulates cell survival.

In periodontitis, pathways associated with hemostasis and thrombosis were significantly enriched, including: “Complement and coagulation cascades” (5.2, *p* = 7.9 × 10^−13^) and “Platelet activation” (3.5, *p* = 5.5 × 10^−8^). Also noteworthy is the enrichment of the “lipid metabolism and atherosclerosis” pathway (Lipid and atherosclerosis, 2.8, *p* = 7.5 × 10^−8^), which may indicate common molecular mechanisms with the atherosclerotic process. In addition, in periodontitis, a higher enrichment of pathways associated with leukocyte transendothelial migration was observed (Leukocyte transendothelial migration, 4.4 vs. 3.0 in peri-implantitis).

#### 3.2.2. Downregulated Genes

**Figure 2:**
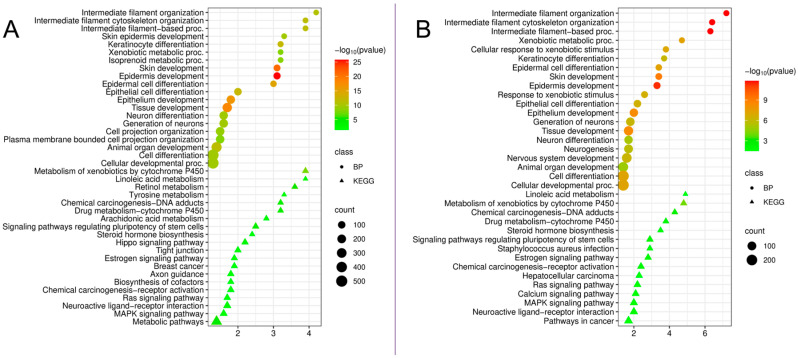
(**A**). Functional enrichment analysis by biological processes (BPs) and KEGG pathways of downregulated DEGs in peri-implantitis compared to the control. (**B**). Functional enrichment analysis by biological processes of downregulated DEGs in periodontitis compared to controls.

Analysis of downregulated DEGs revealed other biological processes that were suppressed in both diseases (Figure 2).

In both conditions, the most significant suppression was observed in cytoskeletal organization processes, with these changes being significantly more pronounced in periodontitis. “Intermediate filament organization”: peri-implantitis—4.2 (*p* = 1.7 × 10^−11^), periodontitis—7.2 (*p* = 1.5 × 10^−12^). “Intermediate filament cytoskeleton organization”: 3.9 (*p* = 3.0 × 10^−12^) and 6.4 (*p* = 1.5 × 10^−12^), respectively. “Intermediate filament-based processes”: 3.9 (*p* = 3.9 × 10^−12^) and 6.3 (*p* = 1.5 × 10^−12^), respectively. Intermediate filaments, particularly keratin filaments, are key structural components of epithelial cells that ensure the mechanical integrity of the gingival epithelial barrier. Significant downregulation of genes involved in their organization may indicate cytoskeletal disorganization and impaired barrier function of the epithelium in both diseases. However, in periodontitis, these changes are likely to be more severe.

Analysis of KEGG pathways for downregulated genes revealed significant suppression of metabolic processes in both diseases, which may indicate impaired detoxification mechanisms and lipid metabolism. “Metabolism of xenobiotics by cytochrome P450”: peri-implantitis—3.9 (*p* = 2.6 × 10^−8^), periodontitis—4.8 (*p* = 1.1 × 10^−4^). “Linoleic acid metabolism”: peri-implantitis—3.9 (*p* = 2.1 × 10^−3^), periodontitis—4.9 (*p* = 2.7 × 10^−2^). “Arachidonic acid metabolism”: peri-implantitis—2.8 (*p* = 4.8 × 10^−3^). “Retinol metabolism”: peri-implantitis—3.6 (*p* = 4.2 × 10^−6^). “Drug metabolism—cytochrome P450”: peri-implantitis—3.2 (*p* = 5.5 × 10^−5^), periodontitis—3.8 (*p* = 8.9 × 10^−3^). “Chemical carcinogenesis—DNA adducts”: peri-implantitis—3.2 (*p* = 5.1 × 10^−5^), periodontitis—4.3 (*p* = 3.1 × 10^−3^). In periodontitis, suppression of pathways associated with signaling cascades involved in cell proliferation and differentiation has also been identified, as follows. “Signaling pathways regulating pluripotency of stem cells”: 2.9 (*p* = 8.4 × 10^−3^). “Ras signaling pathway”: 2.2 (*p* = 2.2 × 10^−2^). “MAPK signaling pathway”: 2.0 (*p* = 2.2 × 10^−2^). This may reflect impaired tissue regenerative potential in chronic periodontitis.

Thus, analysis of downregulated genes identified three key classes of suppressed processes in both diseases: (1) disruption of cytoskeletal organization and epithelial barrier integrity, including disorganization of intermediate filaments and suppression of epithelial differentiation; (2) metabolic abnormalities, including suppression of cytochrome P450 detoxification systems and lipid metabolism; and (3) disruption of signaling pathways regulating regeneration, observed only in periodontitis. At the same time, periodontitis is characterized by a significant enrichment of these pathways, which may reflect clinical differences between the diseases.

### 3.3. Identification of Key Hub Genes with Upregulated Expression and Construction of a PPI Network

**Figure 3:**
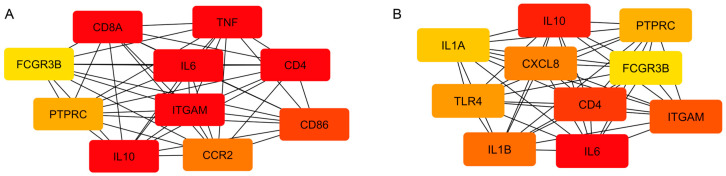
(**A**). The most significant (key hub) upregulated DEGs in peri-implantitis compared to the control group. (**B**). The most significant (key hub) upregulated DEGs in periodontitis compared to the control group. Note: The most significant (key hub) differentially expressed genes were identified in the PPI network using the MCC algorithm in CytoHubba, Cytoscape. The key hub genes are ranked as follows: the most significant genes are highlighted in red, less significant ones in orange, and even less significant ones in yellow.

To identify central regulatory hubs in the pathogenesis of each disease, protein–protein interaction (PPI) networks were constructed and analyzed based on DEGs. For peri-implantitis, the MCC algorithm in the cytoHubba plugin identified the following list of 10 key hub upregulated DEGs: *ITGAM*, *FCGR3B*, *CCR2*, *IL10*, *CD4*, *PTPRC*, *CD8A*, *IL6*, *CD86*, *TNF* (Figure 3A). A similar analysis for periodontitis identified the top 10 key hub upregulated DEGs: *ITGAM*, *CXCL8*, *IL1B*, *FCGR3B*, *PTPRC*, *TLR4*, *CD4*, *IL6*, *IL10*, *IL1A* (Figure 3B). A comparative analysis identified common key hub upregulated DEGs for both diseases: *ITGAM*, *FCGR3B*, *PTPRC*, *CD4*, *IL6*, *IL10*.

### 3.4. Functional Characterization of Key Hub Genes with Upregulated Expression

**Figure 4:**
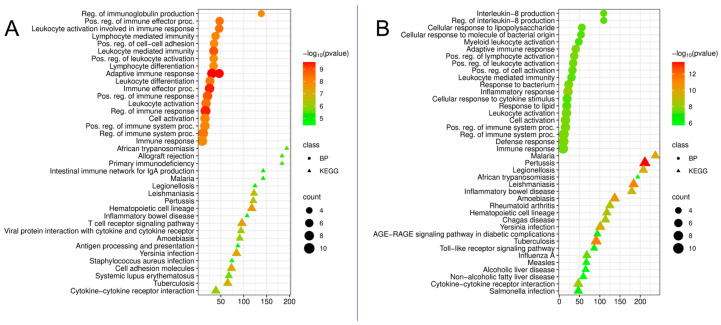
(**A**). Functional enrichment analysis by biological processes and KEGG pathways of the top 10 key hub upregulated DEGs in peri-implantitis compared to the control group. (**B**). Functional enrichment analysis by biological processes and KEGG pathways of the top 10 key hub upregulated DEGs in periodontitis compared to the control.

To identify key regulators of pathogenesis, we analyzed the top 10 key hub upregulated DEGs identified in PPI networks for peri-implantitis and periodontitis. The data confirmed the central role of these genes in immune processes but revealed fundamental differences in the nature of the immune response between the two diseases (Figure 4).

An analysis of the top 10 key hub upregulated DEGs in peri-implantitis (Figure 4A) demonstrated their predominant involvement in adaptive immune mechanisms. The most significant biological processes were: “Regulation of immunoglobulin production,” which may indicate the key role of the humoral (B cell) response; “Adaptive immune response”; “Lymphocyte-mediated immunity”; “Leukocyte activation involved in immune response”; “Lymphocyte differentiation.”

Analysis of KEGG pathways confirmed this picture, revealing a significant enrichment of pathways associated with T cell activation and antigen presentation: “Allograft rejection,” reflecting the activation of mechanisms similar to the rejection of foreign material, which is particularly important in the context of peri-implantitis; “Primary immunodeficiency”; “T cell receptor signaling pathway”; “Antigen processing and presentation”; “Cell adhesion molecules.” The high enrichment of the “graft rejection” pathway is of particular interest, as peri-implantitis develops around the implant. The findings may suggest a possible role for an immune response against implant materials or the biofilm, which is recognized as a foreign agent. Additionally, the enrichment of the “intestinal immune network for IgA production” and “B-cell receptor pathway” pathways is noteworthy, which is consistent with the previously identified enrichment of the B cell pathway in the analysis of all DEGs.

In contrast to peri-implantitis, an analysis of the top 10 key hub upregulated DEGs in periodontitis (Figure 4B) revealed a predominant activation of innate immune mechanisms and the response to bacterial infection. The most significant biological processes were: “Interleukin-8 production,” a key chemotactic factor for neutrophils; “Cellular response to lipopolysaccharide”; “Cellular response to molecules of bacterial origin”, a direct indication of a reaction to bacterial components; “Myeloid leukocyte activation”; “Leukocyte-mediated immunity”; “Response to bacteria”; “Inflammatory response”.

Analysis of KEGG pathways in periodontitis revealed an enrichment of signaling cascades typical of the innate immune response to bacterial pathogens: “Toll-like receptor signaling pathway”—the classic pathway for recognizing pathogen-associated molecular patterns (PAMPs); “AGE-RAGE signaling pathway in diabetic complications”—a pathway linking metabolic stress and inflammation; “Cytokine-cytokine receptor interaction.”

Notably, the term “adaptive immune response” is also frequently observed in periodontitis; however, it was not associated with specific adaptive pathways (such as the B cell receptor pathway or graft rejection).

The data suggest that, despite a similar clinical picture of periodontal tissue inflammation, peri-implantitis and periodontitis have different immunological drivers. Peri-implantitis is characterized by predominant activation of adaptive immunity with a pronounced T cell component and reactions resembling graft rejection, which may be associated with an immune response to implant materials. Periodontitis, in contrast, demonstrates a classic pattern of the innate immune response to bacterial infection with activation of TLR signaling, production of pro-inflammatory cytokines and chemotactic factors (especially IL-8) that recruit neutrophils.

### 3.5. Common Pathways in Atherosclerosis and Inflammatory Periodontal Diseases

To identify common pathogenic signatures between atherosclerosis and inflammatory periodontal diseases, a comparative analysis of DEGs was conducted.

#### 3.5.1. Common DEGs of Atherosclerosis and Peri-Implantitis

**Figure 5:**
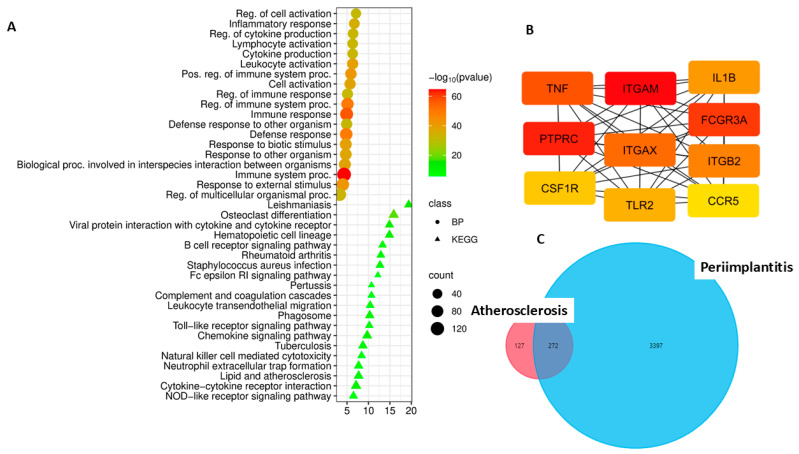
(**A**). Functional enrichment analysis by biological processes and KEGG pathways of common upregulated DEGs in peri-implantitis and atherosclerosis. (**B**). The most significant common upregulated DEGs in peri-implantitis and atherosclerosis, identified in the PPI network using the MCC algorithm in CytoHubba and Cytoscape. The key hub genes are ranked as follows: the most significant genes are highlighted in red, less significant ones in orange, and even less significant ones in yellow. (**C**). Common upregulated DEGs in peri-implantitis and atherosclerosis.

In the atherosclerosis dataset (GSE100927), 399 upregulated DEGs were identified using the selected cutoff criteria. Comparison of these genes with peri-implantitis DEGs revealed 272 upregulated DEGs in both diseases (Figure 5C). Enrichment analysis for the common DEGs revealed their involvement in key immune and inflammatory processes (Figure 5A). The most significant biological processes were: immune response; inflammatory response; leukocyte activation; regulation of cytokine production; and cytokine production, confirming the central role of the immune system in the pathogenesis of both diseases.

Analysis of KEGG pathways revealed a significant enrichment of signaling cascades directly related to both inflammation and vascular pathology: Osteoclast differentiation, a pathway linking inflammation to bone resorption (key to peri-implantitis); leukocyte transendothelial migration, a process critical for the recruitment of immune cells to both periodontal tissue and the vascular wall; chemokine signaling pathway, NF-kappa B signaling pathway—a classic pro-inflammatory cascade; complement and coagulation cascades, a pathway linking inflammation to the hemostatic system; neutrophil extracellular trap formation, the NETosis mechanism involved in the pathogenesis of both diseases; the “Lipids and atherosclerosis” pathway, direct confirmation of the link to lipid metabolism. Particular attention should be paid to the enrichment of the B cell receptor signaling pathway, which is consistent with the previously identified role of adaptive immunity in peri-implantitis and points to a possible role of B cells in atherogenesis.

Among the identified common DEGs, the 10 most significant (key hub) upregulated DEGs were identified (Figure 5B). This list (*PTPRC*, *CCR5*, *ITGAM*, *FCGR3A*, *CSF1R*, *ITGB2*, *TLR2*, *ITGAX*, *TNF*, *IL1B*) includes genes encoding key immune cell receptors and pro-inflammatory cytokines, which may indicate an important role for immunocompetent cells and their effector molecules in the pathogenesis of both atherosclerosis and peri-implantitis.

#### 3.5.2. Common DEGs of Atherosclerosis and Periodontitis

**Figure 6:**
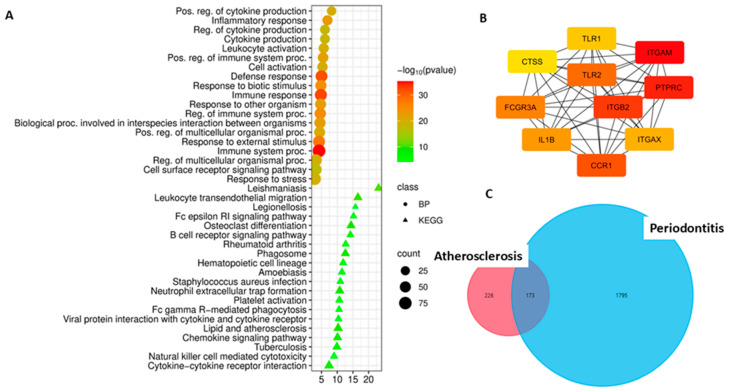
(**A**). Functional enrichment analysis by biological processes and KEGG pathways of common upregulated DEGs in periodontitis and atherosclerosis. (**B**). The most significant common upregulated DEGs in periodontitis and atherosclerosis, identified in the PPI network using the MCC algorithm in CytoHubba and Cytoscape. The key hub genes are ranked as follows: the most significant genes are highlighted in red, less significant ones in orange, and even less significant ones in yellow. (**C**). Common upregulated DEGs in periodontitis and atherosclerosis.

A comparison of atherosclerosis DEGs with periodontitis DEGs identified 173 common upregulated DEGs in both diseases (Figure 6C), indicating significant overlap in transcriptional profiles and the presence of common pathogenic mechanisms.

Enrichment analysis for the 173 common upregulated DEGs revealed their involvement in key immune and inflammatory processes (Figure 6A). The most significant biological processes were: immune response; response to biotic stimulus; inflammatory response; positive regulation of immune system processes; leukocyte activation; and positive regulation of cytokine production. Of particular note is the high significance of the response to biotic stimuli, which may reflect the role of microbial factors in periodontitis and their potential influence on systemic inflammation in atherosclerosis.

Analysis of KEGG pathways revealed a significant enrichment of signaling cascades directly related to inflammation, the immune response, and tissue destruction: Leukocyte transendothelial migration, a process critical for the recruitment of immune cells into both periodontal tissue and the vascular wall; osteoclast differentiation, a pathway linking inflammation to bone resorption (key to periodontitis); phagosome, reflects phagocytosis activity as a mechanism of innate immunity; neutrophil extracellular trap formation, the NETosis mechanism involved in the pathogenesis of both diseases; platelet activation, a pathway linking inflammation to platelet activation, which is important for atherothrombosis; Fc gamma R-mediated phagocytosis, a mechanism of antibody-dependent phagocytosis; Fc epsilon RI signaling pathway, a signaling pathway associated with mast cell activation; natural killer cell-mediated cytotoxicity, a mechanism of Natural killer (NK) cell cytotoxicity; B cell receptor signaling pathway, which may indicate possible involvement of the adaptive immune system; chemokine signaling pathway, a key mechanism of leukocyte chemotaxis; cytokine–cytokine receptor interaction, a central network of intercellular signaling; and “Lipids and atherosclerosis” pathway, which may confirm links to lipid metabolism.

Among the 173 common upregulated DEGs, the 10 most significant DEGs were identified (*CCR1*, *TLR2*, *PTPRC*, *CTSS*, *ITGAX*, *TLR1*, *ITGB2*, *FCGR3A*, *IL1B*, *ITGAM*), with the highest degree of differential expression and a central position in protein–protein interaction networks (Figure 6B). This list includes genes encoding key receptors on immune cells and pro-inflammatory molecules, which may indicate the important role of immunocompetent cells and their effector functions in the pathogenesis of both atherosclerosis and periodontitis.

### 3.6. Cell Type Enrichment Analysis (Immune Deconvolution)

**Figure 7:**
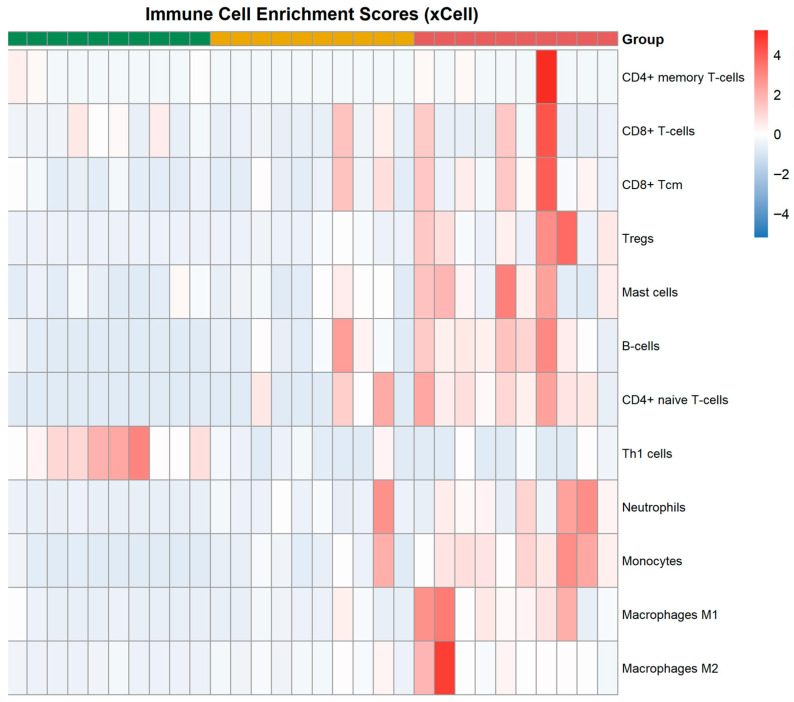
Heat map of relative immune cell abundance (xCell). Rows represent cell types; columns represent samples. The color scale reflects normalized values (from low—blue to high—red). The legend at the top shows the sample groupings: Healthy (green), periodontitis (gold), peri-implantitis (red).

To quantitatively assess the relative abundance of immune cells, an analysis was performed using the xCell algorithm, which enabled the assessment of the representation of 64 cell types in each sample of inflammatory periodontal disease data. The results for key subpopulations of adaptive and innate immunity in periodontitis and peri-implantitis are presented in Table 1 and Figure 7.

xCell analysis revealed a significant increase in B cells in patients with peri-implantitis compared to healthy controls (0.176 ± 0.110 vs. 0.005 ± 0.013, *p* = 0.001), whereas in periodontitis, only a trend toward an increase was observed (0.077 ± 0.108 vs. 0.005 ± 0.013, *p* = 0.064). Among T cell subpopulations, peri-implantitis was associated with a significant increase in CD4+ naive T cells (0.023 ± 0.013 vs. 0.005 ± 0.013, *p* < 0.001), CD8+ central memory T cells (Tcm) (0.034 ± 0.036 vs. 0.004 ± 0.006, *p* = 0.030), and regulatory T cells (Tregs) (0.014 ± 0.016 vs. 0, *p* = 0.020). In periodontitis, these changes did not reach statistical significance. At the same time, a significant decrease in Th1 cells was observed in both periodontitis (0.007 ± 0.008 vs. 0.041 ± 0.024, *p* = 0.001) and peri-implantitis (0.006 ± 0.009 vs. 0.041 ± 0.024, *p* = 0.001).

Analysis of innate immune cells showed that neutrophils (*p* = 0.013), monocytes (*p* < 0.001), M1 macrophages (*p* = 0.008), and dendritic cells (*p* = 0.001) were significantly elevated in peri-implantitis compared to healthy controls. M2 macrophages did not reach statistical significance in peri-implantitis (*p* = 0.062), but were significantly elevated in periodontitis (*p* = 0.005). In periodontitis, only a trend toward increased levels of neutrophils, monocytes, M1 macrophages, and dendritic cells was observed (*p* = 0.064–0.155). Figure 7 shows a heat map illustrating the relative abundance of all types of immune cells in each sample. The peri-implantitis samples are characterized by the highest values for most immune cells.

Thus, xCell deconvolution of transcriptomic profiles revealed that peri-implantitis is characterized by marked activation of both adaptive (B cells, CD4+ naive T cells, CD8+ Tcm, Tregs) and innate (neutrophils, monocytes, macrophages, dendritic cells) immunity. In periodontitis, only trends toward an increase in these cells were observed, with the exception of a decrease in Th1 cells. The key difference between peri-implantitis and periodontitis lies in the more significant and statistically significant activation of both adaptive and innate immunity in peri-implantitis. The results of xCell deconvolution generally align with the functional enrichment data. Consistent with the KEGG analysis, which identified enrichment of the B cell receptor pathway in peri-implantitis, xCell demonstrated a significant increase in B cells in this group (*p* = 0.0014). In periodontitis, where the B cell pathway was not enriched, xCell showed only a trend toward increased B cells (*p* = 0.064).

With regard to innate immunity, despite the identified KEGG enrichment of the TLR pathway and the response to LPS in periodontitis, xCell showed a statistically non-significant trend toward increased neutrophils and macrophages in this group (*p* = 0.064), which may reflect differences between signaling pathway activation and actual cell counts. In contrast, in peri-implantitis, where the TLR pathway was also activated, xCell detected a significant increase in innate immune cells (*p* = 0.0014), indicating more pronounced immune activation.

### 3.7. Diagnostic Significance of Key Genes (ROC Analysis)

To assess the ability of the identified hub genes to differentiate peri-implantitis from healthy tissue, an ROC analysis was performed on the independent dataset GSE33774, which was not used during the DEG identification phase. To validate the diagnostic value of periodontitis hub genes, a large independent dataset, GSE10334 (183 periodontitis samples, 64 controls), was used.

#### 3.7.1. Genes Specific to Peri-Implantitis and Periodontitis

Peri-implantitis. ROC analysis confirmed the diagnostic significance of the identified hub genes. *ITGAM* and *FCGR3A* demonstrated the highest diagnostic accuracy (AUC = 0.964, 95% CI: 0.857–1.000 and 0.839–1.000, respectively; *p* = 0.003 for both). *CD4* and *PTPRC* showed high diagnostic accuracy (AUC = 0.893, 95% CI: 0.679–1.000, *p* = 0.013). IL6 also demonstrated good diagnostic significance (AUC = 0.857, 95% CI: 0.625–1.000, *p* = 0.024). Genes with moderate diagnostic significance: *CD86* (AUC = 0.839, 95% CI: 0.571–1.000, *p* = 0.032) and IL10 (AUC = 0.821, 95% CI: 0.536–1.000, *p* = 0.043). *CCR2*, *TNF*, and *CD8A* showed no statistically significant differences between groups (*p* > 0.05 for all).

Periodontitis. The highest AUCs were demonstrated by *FCGR3B* (AUC = 0.860, *p* < 0.001), *IL1B* (AUC = 0.828, *p* < 0.001), *CD4* (AUC = 0.808, *p* < 0.001), and *CXCL8* (AUC = 0.783, *p* < 0.001). Also significant were *IL6* (AUC = 0.729), *TLR4* (AUC = 0.713), *IL10* (AUC = 0.664), *PTPRC* (AUC = 0.562, *p* = 0.011), and *ITGAM* (AUC = 0.554, *p* = 0.004). *IL1A* did not reach statistical significance (AUC = 0.470, *p* = 0.090).

#### 3.7.2. Genes Shared with Atherosclerosis as Potential Markers of Systemic Inflammatory Risk

Since a significant overlap between the transcriptional profiles of peri-implantitis and periodontitis and those of atherosclerosis had previously been identified (272 and 173 shared genes, respectively), we assessed the diagnostic value of these shared genes for identifying not only local inflammation but also potential systemic risk. To assess the ability of the identified hub genes to differentiate peri-implantitis from healthy tissue, an ROC analysis was performed on the independent GSE33774 dataset. To validate the diagnostic significance of genes shared with atherosclerosis in periodontitis, a large independent dataset, GSE10334 (183 periodontitis samples, 64 controls), was used.

Peri-implantitis. On the independent dataset GSE33774, 9 out of 10 hub genes associated with atherosclerosis demonstrated high diagnostic accuracy in distinguishing between peri-implantitis and healthy controls. The highest AUC values were observed for *FCGR3A* and *ITGAM* (AUC = 0.964 for both), as well as *CSF1R* (AUC = 0.946). *PTPRC*, *IL1B*, *ITGAX* (AUC = 0.893 for all), and *TLR2* (AUC = 0.875) also demonstrated high diagnostic value. *ITGB2* (AUC = 0.821) and *CCR5* (AUC = 0.696) showed moderate discriminatory ability. *TNF* (AUC = 0.679) did not reach statistical significance. The obtained data confirm the central role of genes associated with the monocyte-macrophage lineage (*CSF1R*, *FCGR3A*, *ITGAM*), as well as T cell markers (*PTPRC*, *CD4*—in the analysis of specific genes) in the pathogenesis of peri-implantitis and its molecular overlap with atherosclerosis.

Periodontitis. Eight out of ten genes showed statistically significant diagnostic value (*p* < 0.001 for most). The highest discriminatory ability was demonstrated by *FCGR3A* (AUC = 0.860), *IL1B* (AUC = 0.828), and *CCR1* (AUC = 0.817). High AUC values were also obtained for *ITGB2* (0.769), *CTSS* (0.734), *TLR1* (0.701), *TLR2* (0.685), and *ITGAX* (0.640). The *PTPRC* and *ITGAM* genes did not reach statistical significance (*p* > 0.05).

## 4. Discussion

The comparative transcriptomic analysis revealed both common and specific molecular mechanisms in the pathogenesis of peri-implantitis and periodontitis, and also identified significant overlap between their transcriptional profiles and those of atherosclerosis.

The data confirm that peri-implantitis and periodontitis are characterized by pronounced activation of inflammatory programs, which is consistent with current understanding of their pathogenesis [40]. However, the key finding of the study was the identification of differences in immune mechanisms—the activation of adaptive immunity in peri-implantitis and innate immunity in periodontitis. These differences may underlie the clinical features of the disease course.

The current study has demonstrated that adaptive immune mechanisms are likely involved in the pathogenesis of peri-implantitis. Analysis of the top-10 hub genes revealed pathways associated with immunoglobulin production, T cell activation, and, most importantly, “graft rejection.” This is consistent with the modern concept of osteoimmunology, according to which the immune response plays a critical role in the fate of a dental implant [40].

T-lymphocytes play an important role in peri-implantitis-related inflammation. Previous studies have shown that T lymphocytes (especially CD4+) are the dominant population, and the CD4/CD8 ratio may indicate immune dysregulation compared to periodontitis. Disease progression is associated with an imbalance between pro-inflammatory T-helper subpopulations (Th1/Th17) and regulatory T cells (Treg), leading to increased inflammation and osteoclastogenesis [41,42,43]. The xCell analysis conducted in the present study revealed a significant increase in CD4+ naive T cells (*p* < 0.001), CD8+ central memory T cells (Tcm) (*p* = 0.03), and regulatory T cells (Tregs) (*p* = 0.0231) in peri-implantitis, which may indicate the involvement of specific T cell subpopulations in the pathogenesis. At the same time, a significant decrease in Th1 cells was observed in both periodontitis and peri-implantitis (*p* = 0.0010 and *p* = 0.0009, respectively), which may indicate suppression of Th1-mediated immunity and warrants further investigation in larger studies.

It has previously been shown that peri-implantitis is associated with a significant increase in the number of B cells in the lesions, accompanied by elevated levels of IL-1β, TNF-α, IL-4, and basic fibroblast growth factor [44]. Chen et al. demonstrated that antibody-secreting cells predominate in peri-implantitis tissues, which disappear following successful treatment. This suggests that the absence of tissue-resident memory B cells in healed tissues may be a sign of the peri-implant zone’s vulnerability to bacterial biofilm [21]. Current data on the significant enrichment of B cell pathways are fully consistent with these observations. xCell deconvolution confirmed a significant increase in B cells in the peri-implant area compared to the control group (0.176 ± 0.110 vs. 0.005 ± 0.013, *p* = 0.001), whereas only a trend was observed in periodontitis (0.077 ± 0.108 vs. 0.005 ± 0.013, *p* = 0.064).

In contrast to peri-implantitis, functional enrichment analysis revealed the involvement of innate immune mechanisms in periodontitis. Analysis revealed a predominance of pathways associated with responses to bacterial components, such as lipopolysaccharide (TLR signaling), and the production of chemotactic factors (e.g., IL-8), which is consistent with the classical model of periodontitis as an infectious inflammatory disease initiated by a microbial biofilm. However, xCell did not show a statistically significant increase in neutrophils and macrophages in this group (*p* = 0.064), which may indicate that the activation of signaling pathways can occur without a significant increase in the number of the corresponding cells. Previous studies also confirm that an M1/M2 macrophage imbalance plays a role in pathogenesis; however, this imbalance is more pronounced in periodontitis than in peri-implantitis [45]. An upregulation of the AGE-RAGE pathway was also observed, which may indicate a potential link to metabolic disorders—a finding that is particularly important in the context of the comorbidity of periodontitis and diabetes.

Current results are consistent with the histological studies by Carcuac and Berglundh, which demonstrated differences in the cellular composition of the infiltrate in these two diseases [10]. Peri-implantitis can be characterized as a condition in which an adaptive immune response to the “foreign” implant material is superimposed on chronic inflammation caused by the biofilm, leading to more severe and treatment-resistant inflammation [41]. The identified KEGG pathways associated with the suppression of arachidonic and linoleic acid metabolism may have dual significance. On the one hand, it may reflect impaired synthesis of pro-resolving lipid mediators (resolvins, protectins) involved in the resolution of inflammation. On the other hand, arachidonic acid serves as a substrate for the synthesis of pro-inflammatory leukotrienes [46]. Furthermore, a decrease in cytochrome P450 activity may lead to the accumulation of toxic products and exacerbate oxidative stress, thereby contributing to the maintenance of chronic inflammation.

The most significant finding of this study is the identification of extensive overlap in the transcriptional profiles of both peri-implantitis (272 shared genes) and periodontitis (173 shared genes) with atherosclerosis. The identification of common pathways, such as “leukocyte transendothelial migration,” “cytokine production,” and specifically the “lipids and atherosclerosis” pathway, may support the extensive evidence linking oral pathologies to cardiovascular risk [47,48].

Meta-analyses confirm that periodontal disease is associated with increased intima-media thickness of the carotid arteries and is an independent risk factor for cardiovascular events [49,50,51]. Furthermore, evidence is emerging linking peri-implantitis to more severe coronary artery disease [33]. Current results show that this link has a common molecular basis—systemic inflammation mediated by genes such as *PTPRC*, *ITGAM*, *TLR2*, *TNF*, and *IL1B*. At the same time, the spectra of genes shared with atherosclerosis differ in part between the two dental pathologies, which likely reflects the specific features of their immune mechanisms [52,53].

A comparison of the results of functional enrichment and xCell deconvolution suggests that, in periodontitis, the activation of TLR signaling and the response to lipopolysaccharide occur primarily through the enhancement of intracellular signaling in resident cells, without a significant increase in their numbers. In contrast, in peri-implantitis, TLR signaling is accompanied by a statistically significant increase in the number of innate immune cells (neutrophils, monocytes, macrophages, dendritic cells), indicating a more intense inflammatory response. Similarly, despite the absence of B cell pathway enrichment in periodontitis, xCell revealed a trend toward increased B cells (*p* = 0.064). This may reflect threshold effects related to the sensitivity of the methods. Nevertheless, both methods consistently demonstrate that peri-implantitis is characterized by more pronounced activation of both adaptive and innate immunity compared to periodontitis.

It is important to note that the current study has certain limitations that must be taken into account when interpreting the results. First, the sample size in the GSE223924 dataset is relatively small (10 patients per group). Although this is standard for transcriptomic studies, the results require validation in independent patient cohorts. To mitigate the impact of this limitation, we performed additional validation of the results on independent datasets. However, the independent validation dataset GSE33774 for peri-implantitis was also small (8 controls and 7 cases of peri-implantitis), which may have led to an overestimation of the AUC values in the ROC analysis. Second, transcriptomic analysis reflects expression at the mRNA level, which does not always correlate with protein levels. Proteomic studies and functional experiments are necessary to confirm the role of the identified genes. Third, we lacked information on patients’ clinical characteristics (smoking status, presence of diabetes, current therapy), which may significantly influence transcriptional profiles in both periodontal tissues and the vascular wall. Thus, the presented results should be regarded as a hypothesis that requires confirmation in independent cohorts using additional methods (qPCR, proteomics).

Despite these limitations, the results obtained have important clinical significance. The identified common genes and pathways may serve as potential biomarkers for assessing the risk of cardiovascular complications in patients with periodontal disease and may also be considered promising therapeutic targets for targeted intervention in future studies. Unlike the standard approach, which is limited to analyzing all DEGs, we conducted separate analyses of upregulated and downregulated genes, which enabled us to identify new insights into the transcriptional profiles of peri-implantitis and periodontitis and their shared signaling pathways with atherosclerosis.

## 5. Conclusions

Thus, the present study demonstrates that peri-implantitis is characterized by the activation of both adaptive (B cells, CD4+ naive T cells, CD8+ Tcm, Tregs) and innate (neutrophils, monocytes, M1 macrophages, dendritic cells) immunity, whereas in periodontitis, the activation of innate immunity, as detected at the signaling pathway level, is not accompanied by a statistically significant increase in the corresponding immune cells according to xCell data. Furthermore, the significant molecular similarity between both diseases and atherosclerosis underscores the need for an interdisciplinary approach to managing such patients, focused not only on local treatment but also on the assessment and correction of systemic cardiovascular risk. The identified common genes may provide a basis for further research to develop diagnostic panels for assessing the risk of comorbidity between inflammatory periodontal diseases and atherosclerotic cardiovascular disease.

## Figures and Tables

**Table 1:** Results of the xCell analysis of immune cells.

Type of Cells	Healthy (n = 10)	Periodontitis (n = 10)	Peri-Implantitis (n = 10)	*p*-Value (P vs. H)	*p*-Value (PI vs. H)
Adaptive immunity					
B cells	0.005 ± 0.013	0.077 ± 0.108	0.176 ± 0.110	0.064	0.001
CD4+ naive T cells	0 ± 0	0.010 ± 0.016	0.023 ± 0.013	0.065	<0.001
CD4+ memory T cells	0.0005 ± 0.0009	0 ± 0	0.003 ± 0.007	0.129	0.373
CD8+ T cells	0.009 ± 0.009	0.007 ± 0.014	0.019 ± 0.034	0.740	0.376
CD8+ Tcm	0.004 ± 0.006	0.012 ± 0.019	0.034 ± 0.036	0.224	0.030
Tregs	0 ± 0	0.001 ± 0.002	0.014 ± 0.016	0.062	0.020
Th1 cells	0.041 ± 0.024	0.007 ± 0.008	0.006 ± 0.009	0.001 ↓	0.001 ↓
Th2 cells	0.003 ± 0.006	0.004 ± 0.008	0.004 ± 0.006	0.768	0.856
Innate immunity					
Neutrophils	0.001 ± 0.001	0.021 ± 0.042	0.049 ± 0.050	0.155	0.013
Monocytes	0.002 ± 0.005	0.025 ± 0.037	0.077 ± 0.043	0.084	<0.001
Macrophages M1	0.002 ± 0.005	0.007 ± 0.009	0.048 ± 0.042	0.153	0.008
Macrophages M2	0.001 ± 0.001	0.007 ± 0.006	0.030 ± 0.043	0.005	0.062
Dendritic cells (aDCs)	0.021 ± 0.036	0.057 ± 0.060	0.207 ± 0.125	0.123	0.001
NK cells	0 ± 0	0 ± 0	0 ± 0	0.195	0.297
Mast cells	0.002 ± 0.002	0.003 ± 0.003	0.010 ± 0.008	0.288	0.015
Eosinophils	0 ± 0	0 ± 0	0 ± 0	0.619	0.359
Basophils	0.004 ± 0.005	0.021 ± 0.041	0.015 ± 0.024	0.207	0.187

Note: Data are presented as mean ± standard deviation. *p*-values were calculated using a two-sample *t*-test. Abbreviations: P—periodontitis, PI—peri-implantitis, H—healthy control. ↓—decrease compared to the control group.

## Data Availability

The data presented in this study are available in the Gene Expression Omnibus (GEO) repository at https://www.ncbi.nlm.nih.gov/geo/ (accessed on 11 April 2026), reference numbers GSE223924 and GSE100927. These data were derived from the following resources available in the public domain: GSE223924 (https://www.ncbi.nlm.nih.gov/geo/query/acc.cgi?acc=GSE223924) (accessed on 11 April 2026); and GSE100927 (https://www.ncbi.nlm.nih.gov/geo/query/acc.cgi?acc=GSE100927) (accessed on 11 April 2026).
